# E-cigarette effects on vascular function in animals and humans

**DOI:** 10.1007/s00424-023-02813-z

**Published:** 2023-04-21

**Authors:** Andreas Daiber, Marin Kuntic, Matthias Oelze, Omar Hahad, Thomas Münzel

**Affiliations:** 1 Department of Cardiology 1, University Medical Center of the Johannes Gutenberg University, Langenbeckstr. 1, 55131 Mainz, Germany; 2 German Center for Cardiovascular Research (DZHK), Partnersite Rhine-Main, Mainz, Germany

**Keywords:** E-cigarette vaping, Tobacco cigarettes, Endothelial dysfunction, Oxidative stress, Inflammation

## Abstract

Smoking tobacco cigarettes is a significant (cardiovascular) health risk factor. Although the number of tobacco cigarette users declined over the last decades, shisha smoking and e-cigarette vaping partially compensated for this health benefit. E-cigarettes may create highly addicted dual users (vaping and smoking). E-cigarettes seem not to represent a healthier alternative to tobacco smoking, although they may be less harmful. E-cigarette vaping causes oxidative stress, inflammation, endothelial dysfunction, and associated cardiovascular sequelae. This is primarily due to a significant overlap of toxic compounds in the vapor compared to tobacco smoke and, accordingly, a substantial overlap of pathomechanistic features between vaping and smoking. Whereas the main toxins in vapor are reactive aldehydes such as formaldehyde and acrolein, the toxic mixture in smoke is more complex, comprising particulate matter, reactive gases, transition metals, volatile organic compounds, and N-nitrosamines. However, it seems that both lifestyle drugs impair endothelial function to a quite similar extent, which may be due to the role of oxidative stress as the central pathomechanism to mediate endothelial dysfunction and vascular damage. Finally, the main selling argument for e-cigarette use that they help to quit smoking and get rid of nicotine addiction may be false because it seems that e-cigarettes instead trigger the opposite—younger entrance age and more frequent use. With our review, we summarize the adverse health impact of tobacco cigarettes and e-cigarettes, emphasizing the detrimental effects on endothelial function and cardiovascular health.

## Introduction

**Fig. 1:**
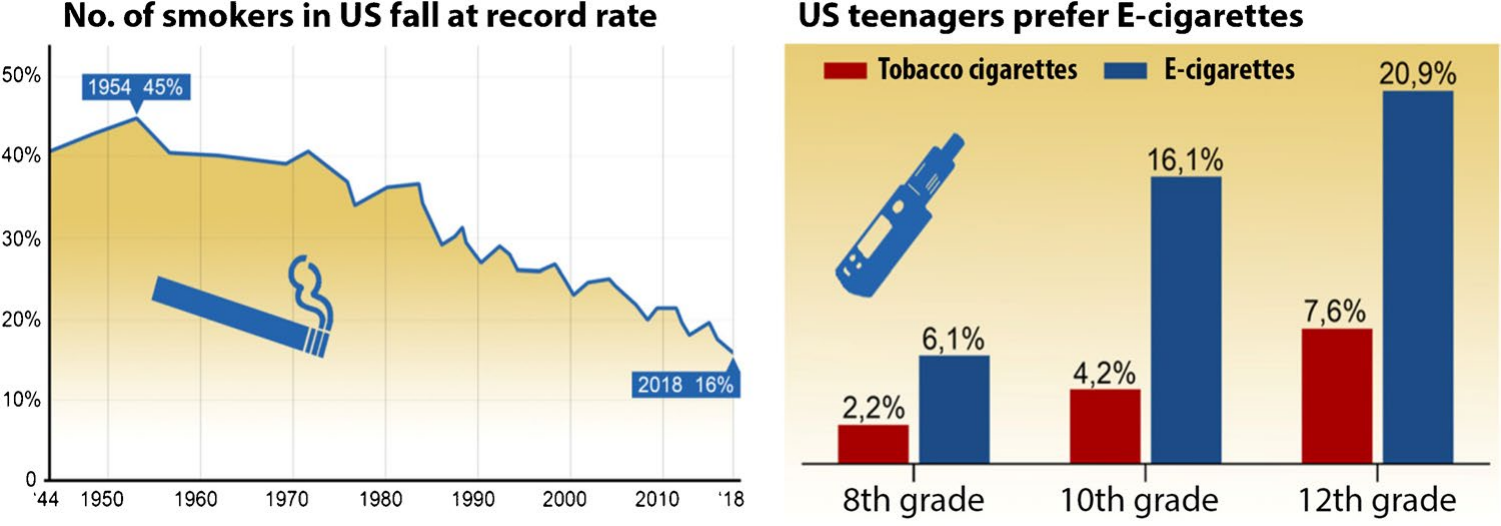
Even though the rate of smokers has declined in the USA for decades, the resulting health advantage is partially offset by the rising number of e-cigarette users, since especially young consumers at schools and colleges favor e-cigarettes. Redrawn from data by Statista: https://www.statista.com/chart/14879/us-smoking-rate-falls-to-record-low/; https://www.statista.com/chart/18633/use-of-cigarettes-and-e-cigarettes-among-us-teens/

The report of the World Health Organization (WHO) from 2015 on the global developments of tobacco smoking starts with the statement that tobacco is a legal drug killing appreciable parts of its users when using it strictly as recommended by the manufacturer or vendor [89]. In 2019, the WHO reported that tobacco consumption kills approximately 50% of its users, and all tobacco-related products together (without e-cigarettes) are responsible for 8 million annual global deaths, and thereof roughly 7 million can be associated with the direct consumption of tobacco and 1.2 million are related to passive smoking [90]. Accordingly, tobacco use is ranked first regarding avoidable health risks and is among the leading risk factors for global morbidity and mortality over the last decades. One of the most recent Global Burden of Disease (GBD) studies even ranked tobacco use second among all health risk factors [16]. The number of tobacco smokers has shown a global reduction during the last 3 decades, mainly due to a lower number of female smokers by 100 million until 2018. However, this decrease in tobacco smokers is offset in part by emerging lifestyle drugs such as e-cigarettes and water pipes (shisha) that are extremely popular among the youth (Fig. 1) [60, 90].

The popularity of e-cigarettes and shisha among the young generation can be attributed to the easy availability of various flavors and the promised healthier alternative to traditional tobacco cigarettes. However, the latter assumption is an erroneous belief upon critically evaluating the available clinical and preclinical data [21]. The use of e-cigarettes in the USA increased ninefold between 2011 and 2015, and the global revenues of e-cigarettes are estimated to reach almost 27 billion US dollars by 2023 [60]. Also, the prevalence of shisha smoking in the general population is 20–25% on average in the USA, UK, and Germany [60]. Although the health risks of e-cigarettes and shisha are regarded as somewhat lower than tobacco cigarettes, there is growing clinical evidence that the use of these emerging luxury foods (for a detailed overview, see [60]) increases the risk of stroke, myocardial infarction, and coronary artery disease.

## Clinical evidence for cardiovascular risk by e-cigarettes

There is substantial evidence for the acute detrimental effects of e-cigarettes and the toxic compounds generated in the vapor on cardiovascular health, primarily by increasing oxidative stress and causing endothelial dysfunction [69]. However, studies addressing the long-term effects of e-cigarettes and cardiovascular risk are limited and controversial. Two large clinical trials showed an association of e-cigarette vaping with a higher risk of myocardial infarction with an odds ratio (OR) of 2.11 (confidence interval (95% CI) 1.14–3.88) [24] and an OR of 1.79 (95% CI 1.20–2.66) [1], however, with varying results for the pooled analysis or when looking at former e-cigarette use. In contrast, a systematic review and meta-analysis concluded that e-cigarettes acutely impair heart rate and blood pressure but still show benefits of switching from tobacco to e-cigarettes [78]. Likewise, the cardiovascular disease burden in former smokers was decreased when switching to e-cigarettes but without clear benefits concerning blood pressure and heart rate [30]. Notably, most e-cigarette users are former tobacco cigarette or dual users, and e-cigarettes may facilitate smoking initiation in individuals who never smoked [56].

The most recent large-scale clinical trials did not report a significantly increased risk of e-cigarette use. A large cohort of 449,092 participants from the US found no association between e-cigarette use and prevalent cardiovascular disease (composite of coronary heart disease, myocardial infarction, or stroke) among individuals who never smoked [66]. In contrast, dual-use (vaping and smoking) was associated with a 36% (odds ratio 1.36, 95% confidence interval 1.18–1.56) higher risk of cardiovascular disease compared with current smokers who never vaped. In a recent longitudinal analysis from Berlowitz et al., it was demonstrated that the risk of incident cardiovascular disease (composite of myocardial infarction or needed bypass surgery, heart failure, other heart condition, or stroke or composite of only myocardial infarction, heart failure, or stroke) did not differ between participants using e-cigarettes exclusively and nonusers [6]. Furthermore, there was a tendency to show that exclusive e-cigarette use was associated with a 30 to 40% lower risk of cardiovascular disease compared to exclusive smoking. There was also no difference in cardiovascular risk when comparing exclusive e-cigarette use with nonuse of cigarettes and e-cigarettes. In contrast, dual use of cigarettes and e-cigarettes was associated with increased cardiovascular disease risk compared with nonuse.

Overall, there is a significant variation in the main results among these clinical studies that may be explained as follows. One study mentioned above represents a meta-analysis of acute interventional and observational studies leading to the conclusion that e-cigarette use increases the risk for cardiovascular disease [78]. Another report represents a controlled trial of switching from combustible tobacco cigarettes to e-cigarettes [30]. The major outcome was that after 1 month of follow-up, flow-mediated dilation was improved. Still, none of the secondary outcomes were improved (e.g., pulse wave velocity, heart rate, circulating inflammation markers). The authors also reported that some subjects continued with dual-use, making this study less reliable. A third study represents a review article, where authors have included animal and human studies [56]. Also, since it is not a systematic review, the authors have some freedom when making the main conclusion. The last trial represents a longitudinal study from a self-reporting cohort [6]. Here, the risk for heart failure and myocardial infarction was elevated but nonsignificantly in the exclusive e-cigarette use group compared to nonsmokers.

As nicotine is a known developmental toxicant (Report of the Surgeon General [83]), e-cigarette consumption during pregnancy is expected to result in risks for the fetus. Only limited observational studies have been conducted in humans [9, 10, 15, 71], and no data are available on cardiovascular development. Animal studies provide more information and point to changes in the offspring’s cardiovascular and pulmonary function (for review, see [35]). Another critical point is the contribution of second-hand vapor effects (similar to the well-established secondhand smoke adverse health effects [88]). These studies on second-hand e-cigarette vapor are scarce and only focus on the chemical composition and prevalence, not on the cardio-pulmonary effects. However, a study showed a correlation between second-hand e-cigarette vapor and asthma events in teenagers [4].

## Oxidative stress and endothelial dysfunction

The pathomechanisms of smoking that led to cardiovascular diseases (CVD) and mortality are complex and only partially characterized. Endothelial dysfunction, most probably mediated by oxidative stress [18], is an early functional parameter reflecting cardiovascular damage in cigarette smokers [12]. Endothelial dysfunction is also considered an early predictor of future cardiovascular events or, in general, poor cardiovascular prognosis [18, 59]. Human endothelial function is frequently determined by flow-mediated dilation (FMD) in the forearm, a technique where the reinitiated blood flow after occlusion (ischemia) triggers endothelial ^•^NO formation and vasodilation by mechanical and reperfusion-dependent stimuli (= hyperemia) [81].

**Fig. 2:**
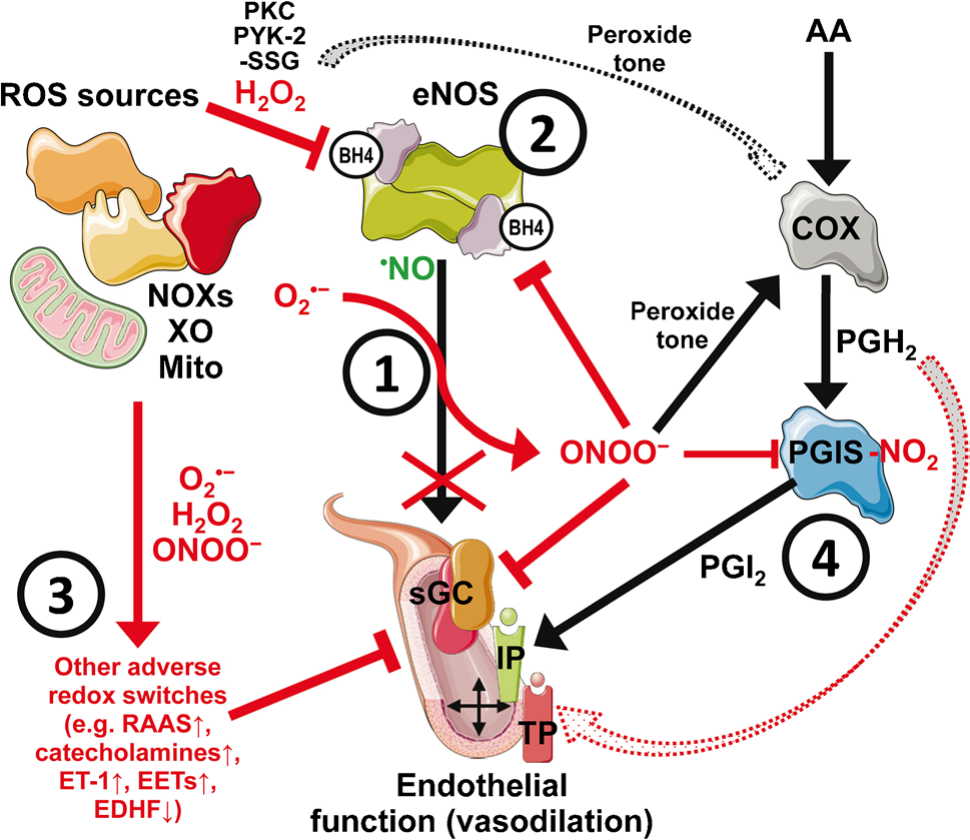
Simplified scheme of redox regulation of vascular tone showing the major adverse redox processes that contribute to endothelial (vascular) dysfunction (personal selection by the authors). Reactive oxygen species (ROS) such as superoxide (O_2_^•−^) and hydrogen peroxide (H_2_O_2_) are formed by the critical ROS sources NADPH oxidases (NOXs), xanthine oxidase (XO), and mitochondrial respiratory chain (Mito). (1) Nitric oxide (^•^NO) from eNOS, a potent vasodilator via activation of the soluble guanylyl cyclase (sGC), is oxidatively depleted by reaction with superoxide anion radicals, yielding the strong oxidant peroxynitrite (ONOO^−^), which causes oxidative inactivation of sGC. (2) eNOS becomes inactivated and uncoupled by various redox mechanisms, e.g., oxidative depletion of tetrahydrobiopterin (BH4) by peroxynitrite or hydrogen peroxide-dependent activation of tyrosine and serine kinases (PYK-2 and PKC) that confer inhibiting phosphorylation of eNOS as well as S-glutathionylation (–SSG) of the reductase domain. (3) Other adverse redox switches are the ROS-triggered activation of the renin–angiotensin–aldosterone system (RAAS), catecholamines such as noradrenalin, endothelin-1 (ET-1), as well as the dysregulated synthesis of epoxyeicosatrienoic acids (EETs) and endothelium-derived hyperpolarizing factor (EDHF). (4) Prostanoid synthesis from arachidonic acid (AA) is dysregulated by ROS-dependent activation of the cyclooxygenase (COX) by providing the peroxide tone, leading to higher production of the endoperoxide prostaglandin H2 (PGH_2_). The latter process causes vasoconstriction via the thromboxane/PGH_2_-receptor (TP), mainly when PGH_2_ accumulates due to peroxynitrite-dependent nitration (–NO_2_) and inactivation of the prostacyclin synthase (PGIS). Thereby, vasodilation by activating the prostacyclin (PGI_2_)-receptor (IP) is lost. Contains images from Servier Medical Art by Servier, licensed under a Creative Commons Attribution 3.0 Unported License

Functional endothelial cells are key to regulating vascular tone, inflammation, vascular growth, platelet aggregation, and coagulation [18, 59]. Generating vasodilators by the endothelium that mediate remarkable antiatherosclerotic and antiaggregatory effects, e.g., nitric oxide (^•^NO) and prostacyclin, is a central mechanism ensuring a healthy vasculature. Endothelial dysfunction represents a hallmark of all cardiovascular diseases and is based on dysregulated biochemical pathways in the endothelium [33]. The pathophysiology underlying endothelial dysfunction is complex, but a higher abundance of reactive oxygen species (ROS) in the vasculature, stemming from ROS sources such as nicotinamide adenine dinucleotide phosphate (NADPH) oxidase, xanthine oxidase, mitochondria, and uncoupled endothelial nitric oxide synthase (eNOS) [18, 29, 59], represents a key pathomechanism of all cardiovascular disease with associated endothelial dysfunction [36, 37]. Few central oxidative stress-mediated pathomechanistic reactions in the vasculature are thought to largely contribute to endothelial dysfunction, blood pressure increases, atherosclerotic changes, and ischemic events (summarized in Fig. 2) [18, 33]:

1. The direct removal of nitric oxide by superoxide anion radicals leads to diminished endothelial-dependent vasodilation [38] and phenotypic change of the vascular environment to a pro-atherothrombotic and vasoconstrictor condition [3], which can be biochemically explained by the diffusion-controlled reaction of ^•^NO with superoxide yielding the highly reactive oxidant peroxynitrite (ONOO^−^) [5].
2. Oxidative uncoupling and inactivation of eNOS by depletion of tetrahydrobiopterin (BH_4_) by peroxynitrite [50, 52], S-glutathionylation of cysteine residues in the reductase domain [14], or adverse phosphorylation by redox activated protein kinases of tyrosines (PYK-2) [55] and serines (PKC) [26, 54]. Uncoupling and inactivation of eNOS are considered highly detrimental to the vasculature [28], and the mentioned “redox switches” are operative in several cardiovascular diseases [77].
3. Reactive oxygen species-mediated activation of vasoconstrictor pathways such as endothelin-1 via induction of its promoter [43, 44] and increases of circulating catecholamines and angiotensin-II [34].
4. Tyrosine nitration and inactivation of prostacyclin synthase by peroxynitrite is another example of how oxidative stress impairs vascular function [91, 92], mainly since peroxynitrite also provides the peroxide tone for cyclooxygenases and thereby promotes the formation of the vasoconstrictors prostaglandin endoperoxide (PGH_2_) and thromboxane [2, 76].

**Fig. 3:**
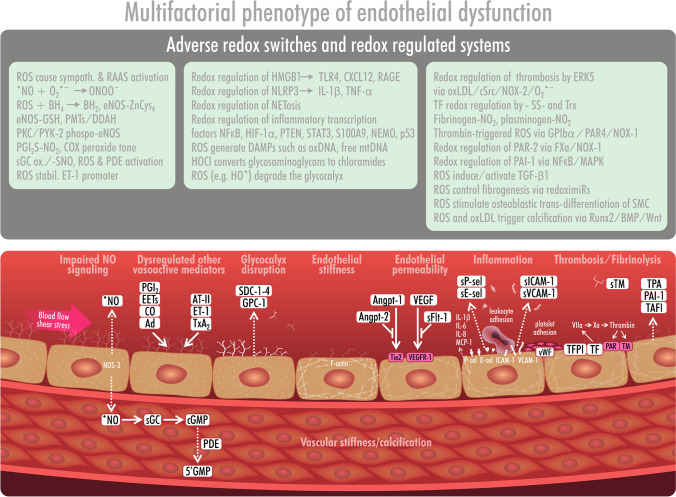
Endothelial dysfunction—a complex multifactorial phenotype. Processes contributing to endothelial (vascular) dysfunction are not limited to the impaired NO-dependent function but also involve dysregulation of numerous other vasoactive mediators: glycocalyx disruption, endothelial (vascular) stiffness, increased endothelial permeability, endothelial inflammation, and alterations in thrombotic or fibrinolytic mechanisms. Known redox switches in these components are shown above these processes and are explained in full detail in the original article. Reused from [17] with permission. © 2020 The Authors. Published by Elsevier Inc

Besides these major oxidative stress-mediated pathways that confer cardiovascular damage, dysregulated fatty acid metabolism and signaling is an essential contributor to impaired vascular/endothelial homeostasis, e.g., by activation of the soluble epoxide hydrolase leading to altered formation of epoxyeicosatrienoic acids (EETs), enhanced thromboxane/prostaglandin endoperoxide H2 (TP) receptor signaling [22, 73], and impaired synthesis of endothelium-derived hyperpolarizing factor (EDHF), which may per se contribute to ROS formation [25, 27]. Finally, many other redox processes are related to the dysregulation of vasoactive mediators, such as glycocalyx modifications, endothelial permeability, inflammation, and thrombosis. Selected other processes are summarized in Fig. 3, providing a more detailed yet incomplete picture of redox regulation of vascular tone. The underlying adverse redox processes are explained in full detail in a previous review [17].

## Impact of e-cigarette vaping on endothelial dysfunction

**Fig. 4:**
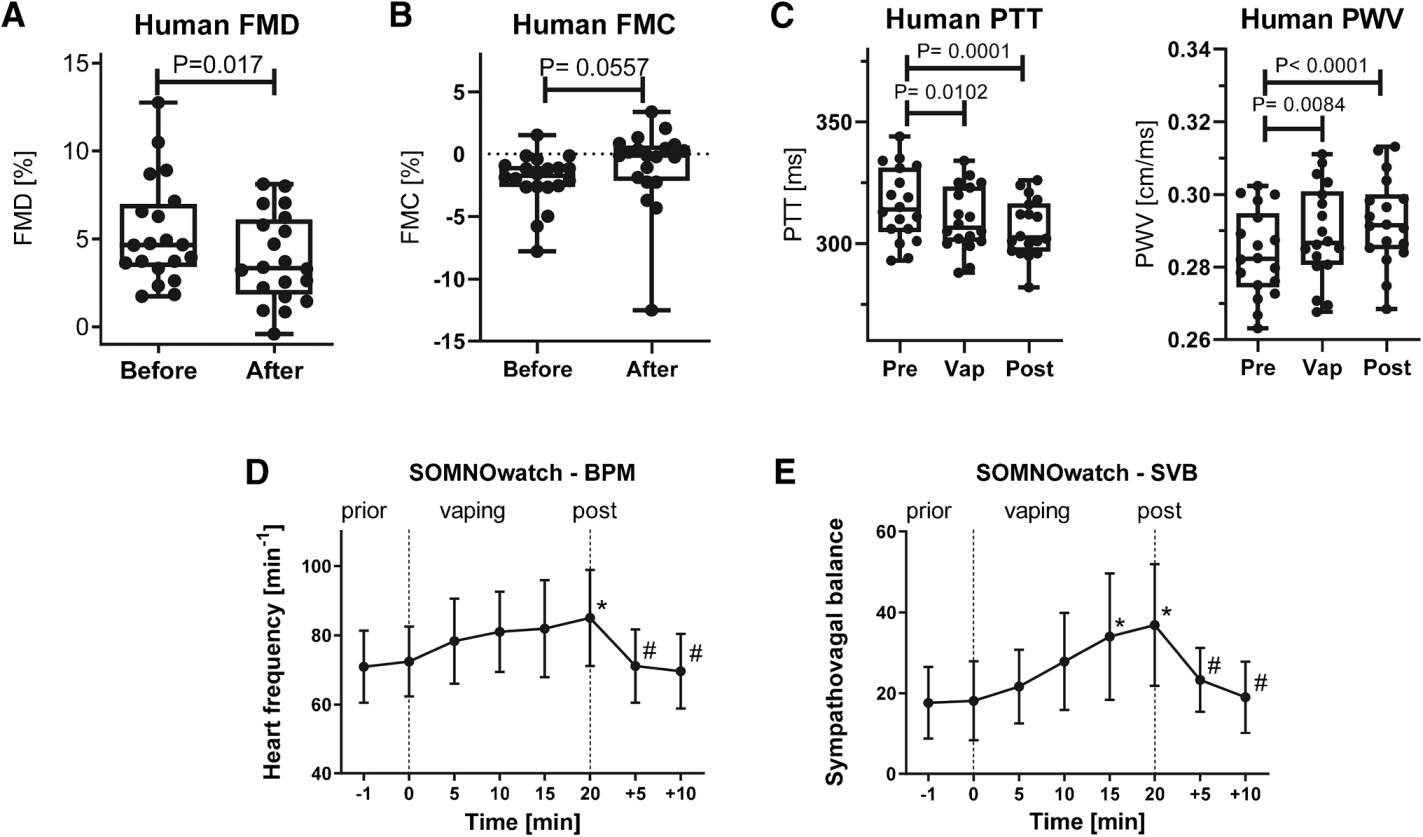
Upper panels: Effects of short-term e-cigarette vapor exposure on humans' cardiovascular function and oxidative stress. Endothelial function in healthy subjects was measured by flow-mediated dilation (FMD) (**A**), low flow-mediated constriction (FMC) (**B**), and arterial stiffness by pulse transit time (PTT)/pulse wave velocity (PWV) (**C**) after a single use of e-cigarette in a cross-over fashion. Data are mean ± SD from *n* = 20 healthy subjects. Lower panels: Polysomnographic (SOMNOwatch™) measurements by fingertip plethysmography and electrocardiogram (ECG) in healthy smokers. The hemodynamic parameters heart frequency (**D**) and sympathovagal balance (**E**) were continuously assessed before, during, and after the e-cigarette vaping session of healthy voluntary smokers using the SOMNOwatch technology. Data are mean ± SD of *n* = 20 healthy smokers prior to, during, and post the vaping session. *, *p* < 0.05 versus *t* = 0 min; #, *p* < 0.05 versus *t* = 20 min. Reused from [47] with permission. Copyright © 2019, Oxford University Press

An inherent problem of the research on e-cigarettes impact on endothelial function is that the published studies focus on short-term effects. There are also problems in comparing different studies due to differences in the methods used to assess endothelial function, the study population (e.g., former, actual, or never smokers), the technical settings of the e-cigarette devices used, as well as liquid composition (nicotine content and flavoring), and the vaping protocol. However, there is a consensus on the potential health risks of e-cigarette use [21], partly by the association of vaping with endothelial dysfunction [60, 78]. A small cohort study conducted a head-to-head comparison of the adverse effects of acute vaping and smoking on endothelial function (measured by FMD), ^•^NO bioavailability, and markers of oxidative stress [11]. No significant difference concerning the adverse effects was found between acute smoking and vaping. A similar trend with somewhat more pronounced effects from tobacco smoke was also seen by others [84]. Also, our studies support that acute e-cigarette vaping leads to endothelial dysfunction (measured by FMD and FMC), enhanced arterial stiffness (PTT and PWV), higher heart rate, and sympathovagal activation in healthy smokers (Fig. 4) [47], which was recapitulated by others using e-cigarette liquid with nicotine but no effect in the absence of nicotine [13]. A study on healthy smokers compared the short-term impact of heat-not-burn devices, e-cigarettes, and tobacco cigarettes and found impaired FMD in all three groups, however, with a particular advantage for the heat-not-burn and e-cigarette devices as compared to tobacco smoking [7].

**Fig. 5:**
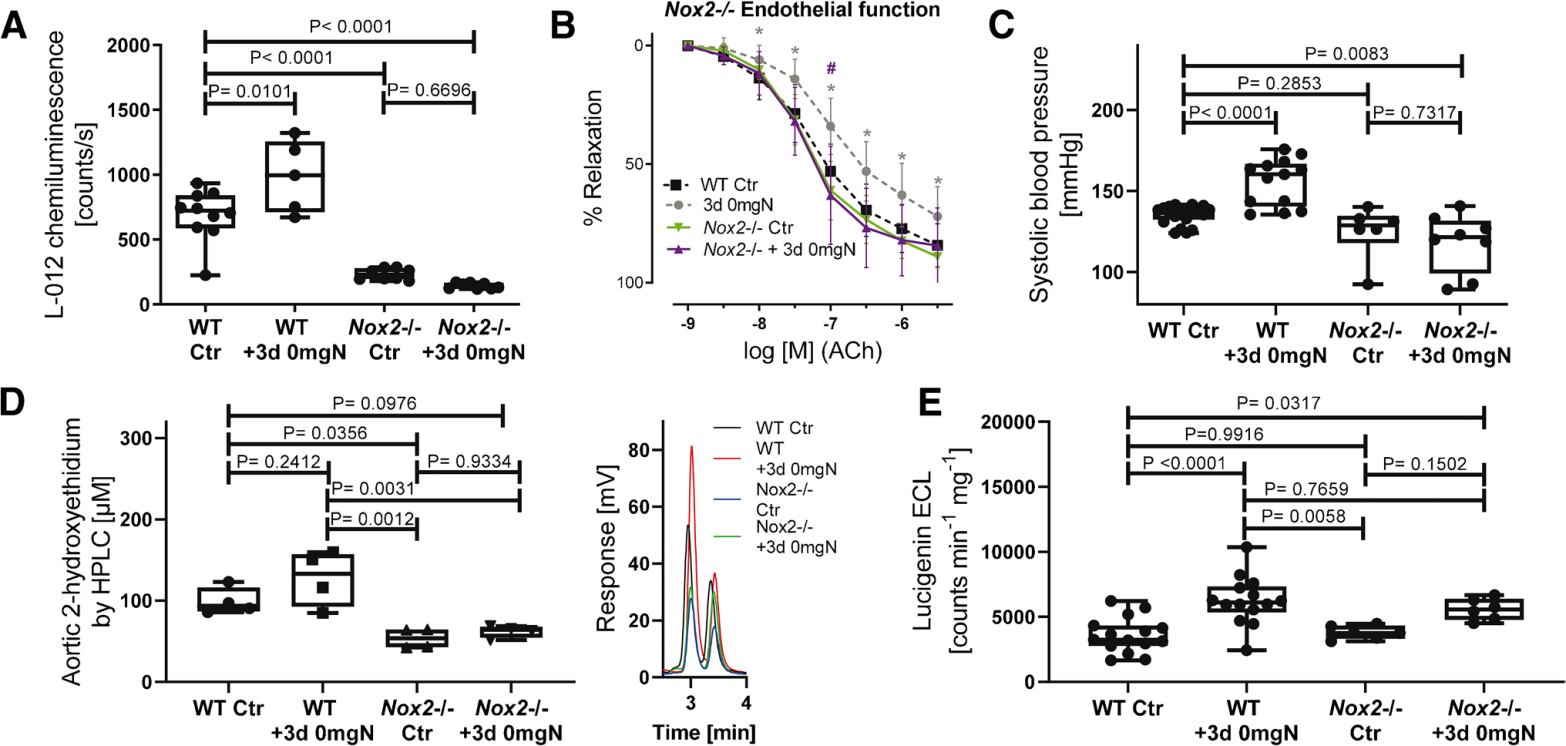
Effects of short-term e-cigarette vapor exposure on vascular function and oxidative stress in *Nox2*^*−/−*^ (*gp91phox*^*−/−*^) versus wild-type mice. **A** E-cigarette vaping caused a more vigorous oxidative burst in the whole blood of wild-type mice, whereas no significant change was observed in the whole blood of *Nox2*^*−/−*^ mice. In addition, the overall stimulated signal was much lower in *Nox2*^*−/−*^ mice. **B** E-cigarette vaping caused endothelial dysfunction in wild-type mice, which was prevented by *Nox2*^*−/−*^. **C** E-cigarette vaping increased systolic blood pressure in the wild type but not in *Nox2*^*−/−*^ mice. **D**, **E** *Nox2*^*−/−*^ knockout prevented e-cigarette vaping-induced increase in aortic ROS/superoxide formation as assessed by HPLC-based quantification of DHE oxidation products and lucigenin-enhanced chemiluminescence (ECL). Representative chromatograms are also shown. Data are presented as boxes (first and third quartiles, line = median) and whiskers (min, max) with jitter plots of single values. *P*-values as indicated except for *, *p* < 0.05 versus unexposed controls. Reused from [47] with permission. Copyright © 2019, Oxford University Press

Animal data showed similar effects on endothelial function by tobacco and e-cigarettes (summarized in [60]). Notably, a study on chronic vaping versus smoking of mice for 8 months indicated comparable impairment of arterial stiffness and endothelial function in isolated vessels by both lifestyle drugs [65]. Short-term vaping of rats using e-cigarette liquid with high nicotine concentrations (JUUL) displayed a substantial degree of endothelial dysfunction that was comparable to impairment of FMD by liquid with regular nicotine concentrations or classic tobacco cigarettes (Marlboro Red) [62, 72]. Our data demonstrated that exposure of mice to e-cigarette vapor for up to 5 days led to substantial endothelial dysfunction, cardio-/cerebrovascular ROS formation, eNOS uncoupling, higher activity of the phagocytic NADPH oxidase (NOX-2), exacerbated endothelin-1 expression, as well as adduct formation of acrolein with proteins [47]. The cardiovascular damage was more evident when liquid without nicotine was used and was prevented by genetic *Nox2* deletion and pharmacological NOX-2 inhibition ex vivo. A deficiency of NOX-2 suppressed the oxidative burst of blood leukocytes that was increased by e-cigarette vapor exposure (Fig. 5A). Also, endothelial dysfunction and elevated systolic blood pressure in e-cigarette vapor-exposed mice were normalized by genetic *Nox2* deletion (Fig. 5B, C). In addition, vascular oxidative stress was increased by e-cigarette vapor exposure (as measured by two independent methods), which was prevented without NOX-2 (Fig. 5D, E). We identified the blockade of endothelin-1-receptor signaling by macitentan and activating the antioxidant and protective transcription factor FOXO3 by bepridil as potential pharmacological targets for mitigating e-cigarette vapor-associated cardiovascular damage. Both drugs prevented e-cigarette vapor-induced endothelial dysfunction, blood pressure increases, and vascular oxidative stress [47].

**Fig. 6:**
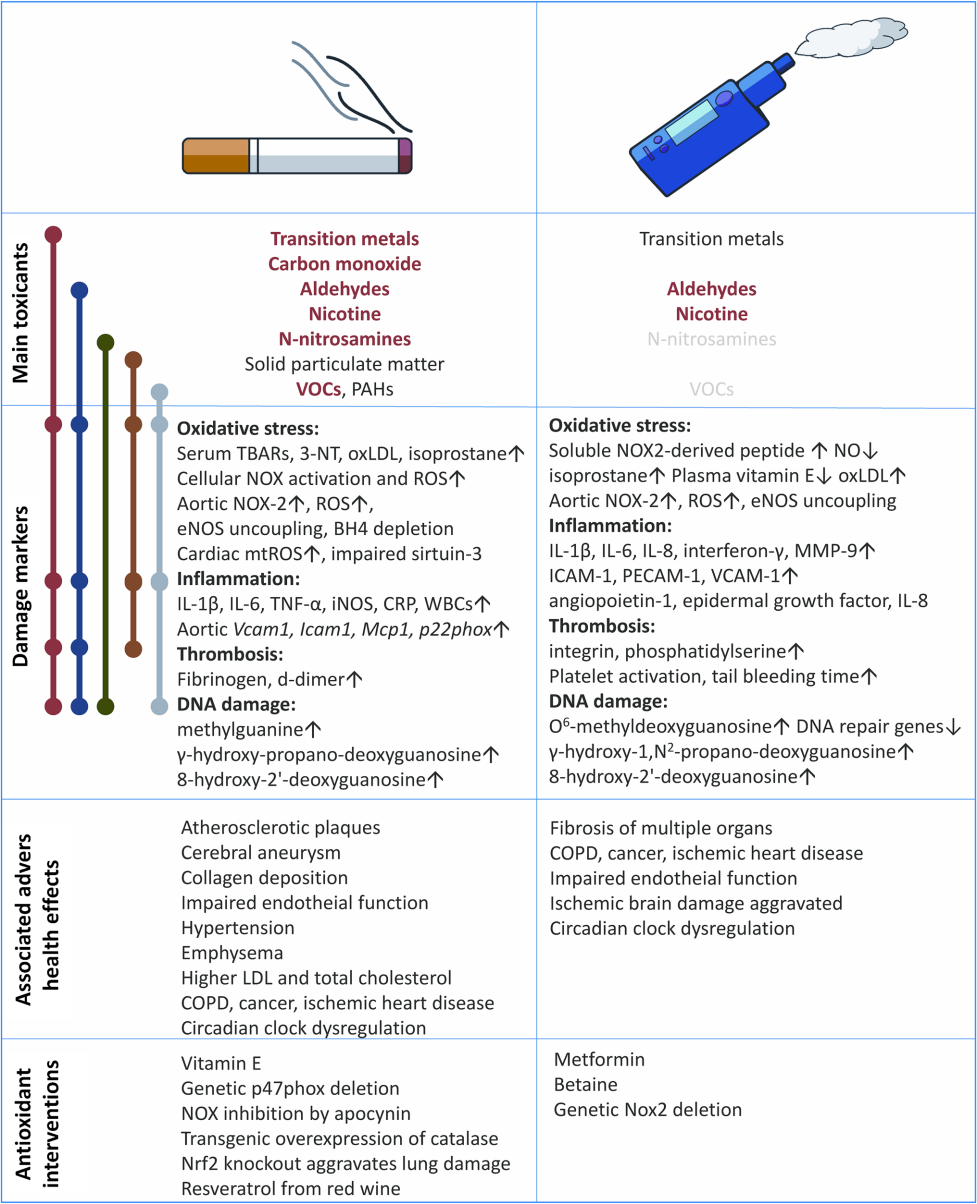
A comparison of the major toxins and adverse health effects of smoking and vaping as summarized from human and animal studies. The major toxicants (red and bold = high quantity, black = intermediate quantity, and gray = trace amounts) for tobacco cigarette smoking and e-cigarette vaping are listed. The molecular link of these toxicants on significant damage markers reported for the different forms of smoking and vaping concerning oxidative stress is shown on the left side. The associated adverse health effects and antioxidant interventions are also shown. Modified from [60] with permission. © 2020, Oxford University Press

Coming back to the above introduced main mechanisms of endothelial/vascular dysfunction, for e-cigarette vapor exposure, it has been shown that more protein tyrosine residues are nitrated, which points toward higher levels of peroxynitrite and oxidative breakdown of nitric oxide, supporting pathomechanisms 1 and 4 [47]. Endothelial dysfunction and nNOS uncoupling support pathomechanism 2 in e-cigarette vapor-exposed mice and sympathovagal activation, and increased endothelin-1 expression and blockade by macitentan points toward pathomechanism 3 in vaping healthy subjects [47]. Others have shown NADPH oxidase (NOX-2) activation, increased vascular ROS formation, higher 3-nitrotyrosine levels, diminished vascular nitric oxide bioavailability, decreased BH4 levels, and eNOS uncoupling by e-cigarette vapor exposure, all of which were associated with endothelial dysfunction and higher blood pressure [23]. Also, exacerbated inflammatory and advanced glycation end-product receptor (RAGE) signaling was observed in e-cigarette vapor-exposed cultured endothelial cells [57]. The reported aggravated thrombogenesis and enhanced platelet function in vapor-exposed mice may point toward impaired prostanoid synthesis [70]. These pathomechanistic features have also been reported for tobacco cigarette smoking by oxidative stress, NADPH oxidase activation, and eNOS uncoupling [20, 79]. Activation of the renin–angiotensin–aldosterone system (RAAS) might be mediated mainly by nicotine [63]. In summary, preclinical studies demonstrated quite similar damage to the vasculature and disturbed endothelial function by vaping and smoking, with oxidative stress and inflammation as critical pathomechanisms (Fig. 6). This was also concluded by previous review articles on e-cigarette vaping that also compared to classical smoking [8, 58, 60, 69].

A study of the retinal microcirculation in e-cigarette users showed that foveal vascular density was significantly decreased and foveal avascular zone area was increased [45]. A study in mice demonstrated a change in regulating several genes associated with BBB function in the cerebral microvessels upon e-cigarette exposure [42]. Studies also indicate that large, conduit vessels (determined by, e.g., flow-mediated dilation of the brachial artery) were negatively affected by e-cigarette use [47, 57]. In conclusion, e-cigarettes may impair conduit and resistance artery function. However, we still do not know the exact mechanisms that explain the interplay between macro- and micro-vasculature in the setting of e-cigarette exposure.

## Toxic compounds and pathomechanisms

The pathomechanisms of e-cigarette-induced cardiovascular damage are far less well characterized than those of tobacco smoking. Also, clinical evidence is less substantial for e-cigarettes than for tobacco use. Nevertheless, the formation of ROS (oxidative stress), inflammation, and endothelial function impairment represent central players mediating the harmful effects of e-cigarette vaping. Impaired endothelial function caused by tobacco cigarette use was already reported in 1993 [12] that was improved by acute administration of vitamins, providing indirect proof of a role for oxidative stress in smoking-mediated endothelial dysfunction [41]. Different methods measured a quite similar impairment of endothelial function by e-cigarette vapor. Vascular damage and endothelial dysfunction caused by adverse lifestyle risk factors, such as smoking and vaping, can be best explained by an appreciable overlap of toxic compounds in the smoke and the vapor (Fig. 6). A list of the known toxic compounds in the smoke of combustible cigarettes is presented in [75], whereas the toxins in e-cigarette vapor and liquid are discussed in [67]. However, the profile of toxic compounds in vapor and smoke varies as the underlying principles vary: smoke is formed by burning tobacco, which produces much higher temperatures than in the heating coil of an e-cigarette during the vaporization process [60]. Pyrolysis creates more harmful and reactive compounds than the relatively moderate temperatures reached during vapor generation.

Nevertheless, it is known that temperatures of > 200 °C can be easily achieved during the generation of the vapor, which is enough to cause fragmentation and oxidation of the main constituents in the liquid, propylene glycol and glycerin, to form reactive aldehydes and ketones [53]. Fragmentation and oxidation of e-cigarette liquid components depend not only on the heating temperature but also on the catalytic properties of the metal surface in the e-cigarette heating element. In commercial liquids, nicotine and flavors lead to a more complex abundance of toxins since these additives can generate secondary toxins during the heating process. We prevented these further complications in our study by only comparing the health effects of the primary liquid with or without nicotine without any flavoring compound [47]. The toxicity of tobacco smoke is mainly determined by transition metals, carbon monoxide and other toxic gases, N-nitrosamines, solid particulate matter (carbon/soot particles), volatile organic (VOCs, e.g., benzene), and polycyclic aromatic hydrocarbons (PAHs, e.g., benzo[a]pyrene) [19, 60] (Fig. 6). In contrast, the most critical toxins in e-cigarette vapor are reactive aldehydes such as formaldehyde and acrolein [31, 64, 80, 82] and low amounts of transition metals. However, N-nitrosamines and VOCs were only detected in trace concentrations in the vapor (see summary in [60]).

The above-mentioned toxic compounds in e-cigarette vapor are potent inducers of oxidative stress and inflammatory reactions with subsequent endothelial dysfunction and progression of atherosclerosis, all of which will increase the cardio/cerebrovascular risk. Our mouse study demonstrated that the reactive aldehydes, namely formaldehyde and acrolein, are most detrimental in mediating the damaging effects of e-cigarette vapor on the vasculature [47]. Figure 6 shows a substantial overlap of toxins in tobacco smoke and e-cigarette vapor, which explains the partially shared pathomechanisms centered around oxidative stress and inflammation [60]. The primary toxic constituents in tobacco smoke, such as particulate matter, toxic gases, VOCs, PAHs, and transition metals, were all shown in the past to cause oxidative stress and inflammation in cells and tissues, which represent potent triggers of endothelial dysfunction and vascular damage. Accordingly, there is substantial overlap in the damage markers of smoking and vaping and some overlap in the reported significant adverse health effects (Fig. 6). Also, genetic or pharmacological antioxidant interventions show similarities in the health benefits between tobacco smoking and e-cigarette vaping.

## Is e-cigarette vaping a less harmful alternative to tobacco smoking?

This question must be answered by a clear “yes” according to the present scientific knowledge and based on the actual clinical evidence (also summarized in [61]). However, whether e-cigarettes harm human health must also be answered with a clear “yes” [48, 49]. A human field study established that a switch from smoking to vaping leads to a rapid and substantial recovery of endothelial function and reversal of vascular stiffness within 1 month [30], supported by a systematic review and meta-analysis [78]. Another human study showed a health benefit for e-cigarette use compared to tobacco smoking, also regarding oxidative stress markers [11]. It is meanwhile also known that the dramatic epidemic of lung disease and pulmonary mortality among e-cigarette users, reported by the US Center for Disease Control (CDC), Food & Drug Agency (FDA), and US State Health Departments, were due to contaminated additives such as vitamin E acetate and tetrahydrocannabinol (THC) or formation of highly toxic products from these additives by the heating process [60]. A primary concern regarding e-cigarette use is that long-term studies rarely evaluate their health effects in the general population (simply because e-cigarettes were introduced into the market only 10 years ago) [61].

Importantly, even if e-cigarettes are less detrimental than tobacco smoking, it should be considered that the age of entrance for consuming e-cigarettes is generally lower than for tobacco cigarettes [60]. In addition, e-cigarette users often become dual users and also smoke tobacco cigarettes on top of vaping. Also, one of the marketing arguments that e-cigarettes help quit tobacco smoking is hardly tenable. A recent study showed that e-cigarettes are better for smoking cessation than nicotine replacement, as 18% finished at 1-year follow-up in the e-cigarette group and only 9.9% in the nicotine replacement group [39]. However, among those who quit, 80% still used their replacement devices in the e-cigarette group, while only 9% still used nicotine replacements in the comparative group. Finally, vaping is easier in public as others consider it less annoying and harmful concerning passive smoking. In addition, since an e-cigarette is designed to be used for only a single puff at a time (without loss of the remaining tobacco cigarette that was just started), it can be used at any 1-min opportunity, e.g., when waiting for traffic lights, the bus, or the train. Especially the use of e-cigarettes among the youth is a matter of concern (Fig. 1) and could generate in the long-term an entirely new generation of addicted nicotine users and also promote chronic disease in later life due to the low age of entrance, which will ultimately transform to more pronounced loss of healthy life years and disability-adjusted life years and thereby increase the global burden of disease.

Smoking cessation is generally the best intervention for preventing the associated adverse health effects. Whereas the acute improvement of both pulmonary and cardiovascular function by smoking cessation becomes obvious within weeks to months (e.g., dyspnea, cough, phlegm, wheezing, exhaled nitric oxide and markers of inflammation) [85], the normalization of the overall cardiovascular and mortality risk to the level of never smokers may take up to 15 years [74]. Whereas e-cigarettes are mostly advertised as a method to quit smoking, there are also profound health improvements after switching from combustible cigarettes to e-cigarettes. Some beneficial cardiovascular effects were observable from as early as 1 month (e.g., improvement of endothelial function by FMD, vascular stiffness by pulse wave velocity) [30]. In contrast, some other parameters show amelioration after a 1-year follow-up (e.g., high blood pressure) [68]. Notably, a large body of evidence still finds no meaningful difference in cardiovascular parameters after switching from combustible cigarettes to e-cigarettes at multiple time points [51]. However, a detailed study of changes in the cardiovascular, or any other parameter, after e-cigarette cessation is complicated because most people switch from combustible cigarettes to e-cigarettes, so it is impossible to separate the cessation from combustible cigarettes as a confounder. We must wait for more extensive studies that only investigate e-cigarette vapers excluding former cigarette smokers.

## Unanswered key questions

This chapter on unanswered questions is inspired by the position statements of the major cardiovascular societies from Europe and the USA [46, 87]. The primary, still unanswered question concerns the long-term use of only e-cigarettes. Since e-cigarettes have been on the world market for only somewhat longer than a decade, the data on their cardiovascular impact in a lifelong use scenario is missing. Also, any data on > 10 years of use of only e-cigarette effects are scary and not high-quality. The second major set of questions deals with the effects of switching from classic tobacco cigarette smoking to e-cigarette vaping. More high-quality data is needed, especially about the age of the users, duration of switching, cumulative duration of use, intensity of use, and the usefulness of e-cigarettes as a cessation device. The next set of unanswered questions deals with the adolescent use of e-cigarettes. There is only limited high-quality data on the addictiveness and the possibility of e-cigarette users becoming classic tobacco cigarette users, although some studies already assessed this issue [40]. Also, there is a lack of psychological and sociological data on how and why adolescents start to use e-cigarettes. Another point is the lack of data on the cognitive effects of e-cigarette use in adolescents. These data will be hard to obtain since controlled trials in this young population are ethically questionable. Therefore, only observational or acute effect studies exist [32, 86]. So, there is a paucity of data on how the use of e-cigarettes in adolescents impacts cardiopulmonary risks at an older age. The last set of questions deals with the population-wide impact and acceptance of different legislative mechanisms to limit e-cigarette sales and use in adolescents. This is important as a disagreement exists on how the sales and regulations should be implemented and as the possibility to use e-cigarettes as smoking cessation devices should remain open.

## Conclusions

The use of tobacco products causes more than 8 million preventable deaths each year, with a major share of cardiovascular causes. Despite the decreasing numbers of tobacco cigarette smokers in recent years, the health benefits of this decrease are partially compensated by higher user numbers for shisha smoking and e-cigarette vaping in combination with the lower entrance age. Vaping causes measurable health effects and may increase the risk of cardiovascular disease, stroke, and chronic lung diseases—although large international population-based studies with > 1 million participants are still lacking. Therefore, the American Heart Association and other cardiovascular medical societies repeatedly published warnings about these lifestyle drugs. E-cigarette vaping shares pathomechanistic features of tobacco smoking, especially oxidative stress, and inflammation, primarily due to an inevitable overlap of the toxic compounds in smoke and vapor. For both lifestyle risk factors, endothelial dysfunction and cardiovascular damage were reported. Although e-cigarettes seem to be less detrimental as compared to tobacco cigarettes (since vapor contains fewer toxic compounds), only a few long-term human studies are available, which makes it impossible to estimate the health risks associated with vaping devices reliably. These facts and the lower entrance age for e-cigarette use warrant their strict regulation (reviewed in [60]). Whereas, in some countries, the sale of e-cigarettes and liquids is prohibited, e.g., in India, Australia, and Mexico, as well as several South American and Arabian countries (making up 35% of the world population), their sales are still allowed, although regulated, e.g., in Europe, the USA, Canada, and China (making up another 35% of the world population). In the last third of the world, e-cigarette and liquid sales are not regulated at all.

## Data Availability

Not applicable.
